# Supplementary material for: Small Molecule Regulation of CLOCK:BMAL1 DNA Binding Activity
Source: bioRxiv. 2026 Apr 14:2026.04.13.718289. Preprint. [Version 1] doi: 10.64898/2026.04.13.718289 (PMC13104816; doi:10.64898/2026.04.13.718289)
Supplement: Supplement 1 [file media-1.pdf]

Supporting Information

Small Molecule Regulation of CLOCK:BMAL1 DNA Binding Activity

Diksha Sharma[1], Soumendu Boral[2], Ethen West[1], McClain Kressman[1], Irene Franco[1], Sarvind Tripathi[1], Hsiau-Wei Lee[1], Carlos A. Amezcua[3,6], Denize C. Favaro[2], Kevin H. Gardner[2,7,8], Carrie L. Partch[1,4,5]*

[1] Department of Chemistry and Biochemistry, University of California Santa Cruz, Santa Cruz, CA 95064
[2] Structural Biology Initiative, CUNY Advanced Science Research Center, New York, NY 10031
[3] Department of Biochemistry, University of Texas Southwestern Medical Center, Dallas, TX 75390
[4] Center for Circadian Biology, University of California San Diego, La Jolla, CA 92093
[5] Howard Hughes Medical Institute, University of California Santa Cruz, Santa Cruz, CA 95064
[6] Current address: FMC Corporation, Newark, DE, 19711
[7] Department of Chemistry and Biochemistry, City College of New York, New York, NY 10031
[8] Ph.D Programs in Biochemistry, Biology, and Chemistry, CUNY Graduate Center, New York, NY 10016

Correspondence:
Carrie Partch
1156 High St. Mailstop: Chemistry
Santa Cruz, CA 95064
Phone: (831) 459-3905 email: cpartch@ucsc.edu

Co-correspondence:
Kevin Gardner
email: kgardner@gc.cuny.edu

Classification: Biological Sciences; Biochemistry

Keywords: small-molecule screening, circadian rhythms, protein-ligand interactions, DNA binding, NMR spectroscopy, transcription factor

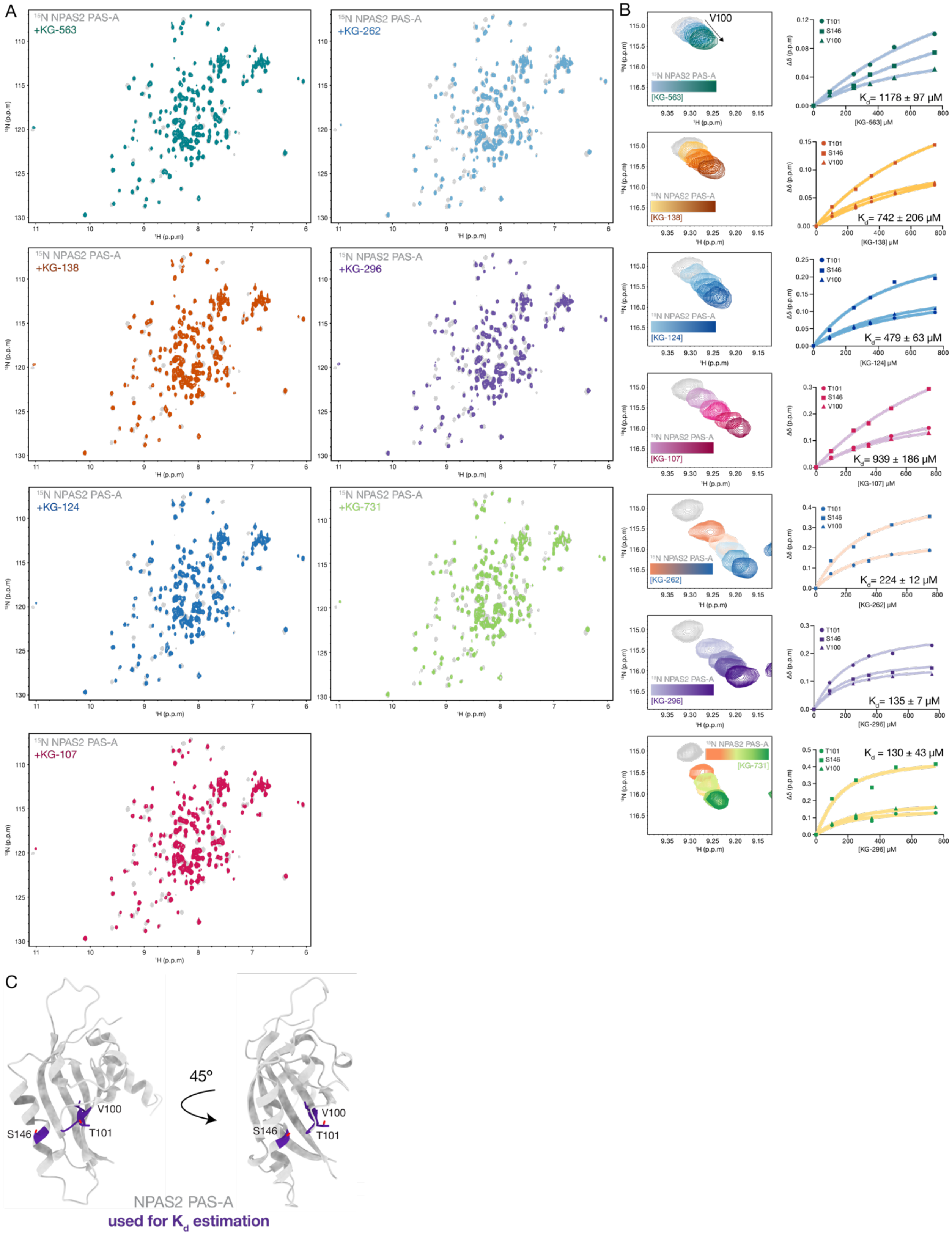

Figure S1: NMR ligand screen of KG library identified 8 positive hits for NPAS2 PAS-A. (A) ^{15}N-^{1}H HSQC of ^{15}N labeled NPAS2 PAS-A (gray) with 500 μM of KG

ligands: KG-563 (dark green), KG-138 (brown), KG-124 (dark blue), KG-107 (pink), KG-262 (blue), KG-296 (purple), KG-731 (green). **(B)** Zoomed view of ^{15}N-^{1}H HSQC of NPAS2 PAS-A V100 (apo, gray) showing dose dependent response of KG ligands (100-750 μM) in ^{15}N labeled NPAS2 PAS-A, KG-563 (light blue to dark green), KG-138 (yellow to brown), KG-124 (light blue to dark blue), KG-107 (light pink to dark pink), KG-262 (peach to dark blue), KG-296 (purple to dark purple), KG-731 (orange to green). **(C)** NPAS2 PAS-A (gray, AlphaFold) showing 3 residues in sticks (purple) used for CSP fitting and apparent K_d estimation.

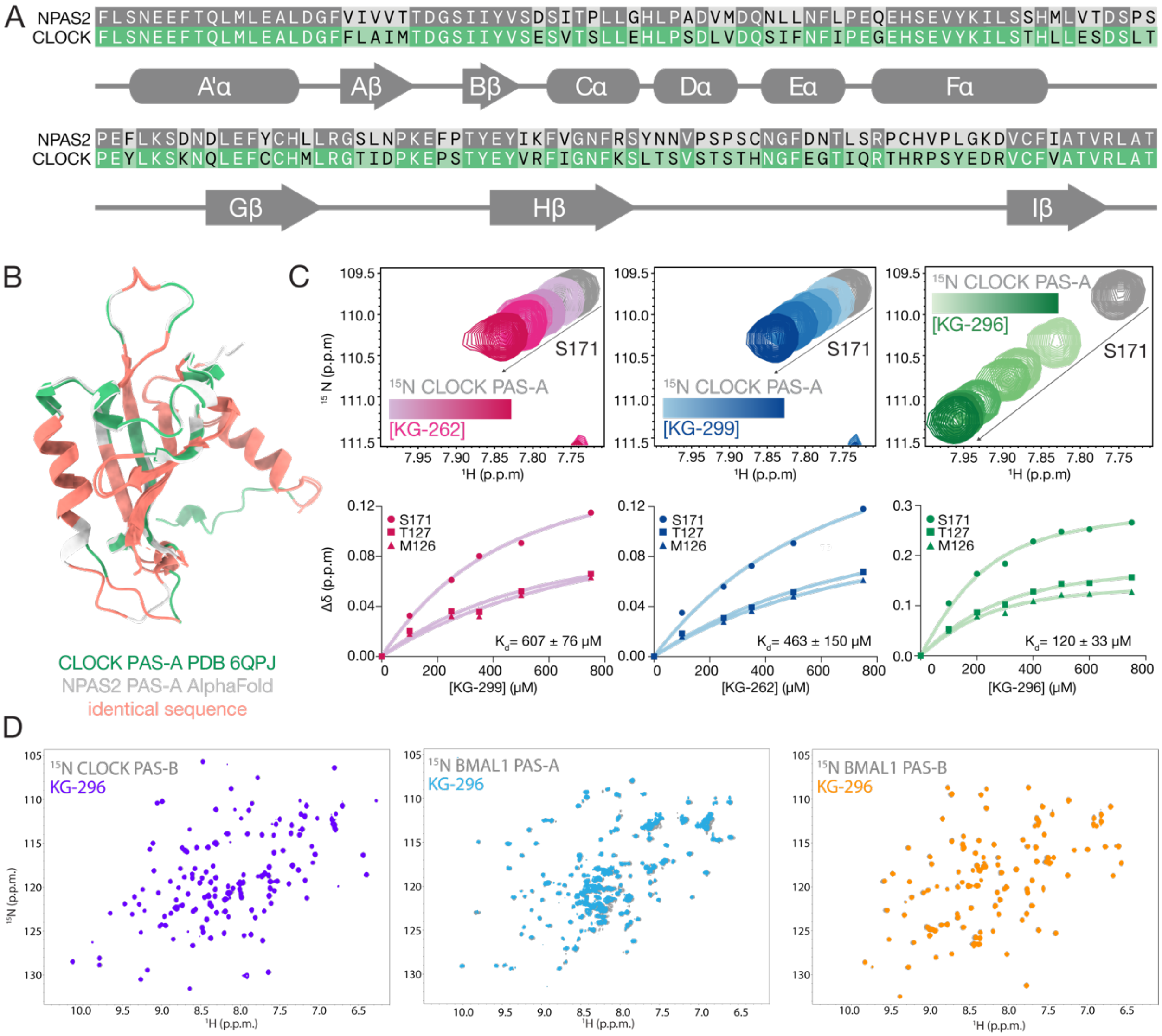

Figure S2: CLOCK PAS-A and NPAS2 PAS-A bind KG compounds similarly. (A) Sequence alignment of human NPAS2 PAS-A (gray) and CLOCK PAS-A (green). Darker shades indicate identical residues. Secondary structures of NPAS2 and CLOCK PAS-A (above). **(B)** Structure overlay of CLOCK (green, PDB 6QPJ) and NPAS2 PAS-A (gray, AlphaFold). Identical residues are shown in salmon. **(C)** Zoomed view of ^{15}N-^{1}H HSQC of S171 in CLOCK PAS-A (apo, gray) showing dose-dependent response (100-750 μM) of KG-262 (pink), KG-299 (blue) and KG-296 (purple). Fitting concentration-dependent CSPs on ^{15}N labeled CLOCK PAS-A to estimate apparent K_d (bottom). **(D)** ^{15}N-^{1}H HSQC spectra of ^{15}N labeled CLOCK PAS-B (gray) with 500 μM KG-296 (violet), ^{15}N BMAL1 PAS-A (gray) with 500 μM KG-296 (sky blue), ^{15}N labeled BMAL1 PAS-B (gray) with 500 μM KG-296 (orange).

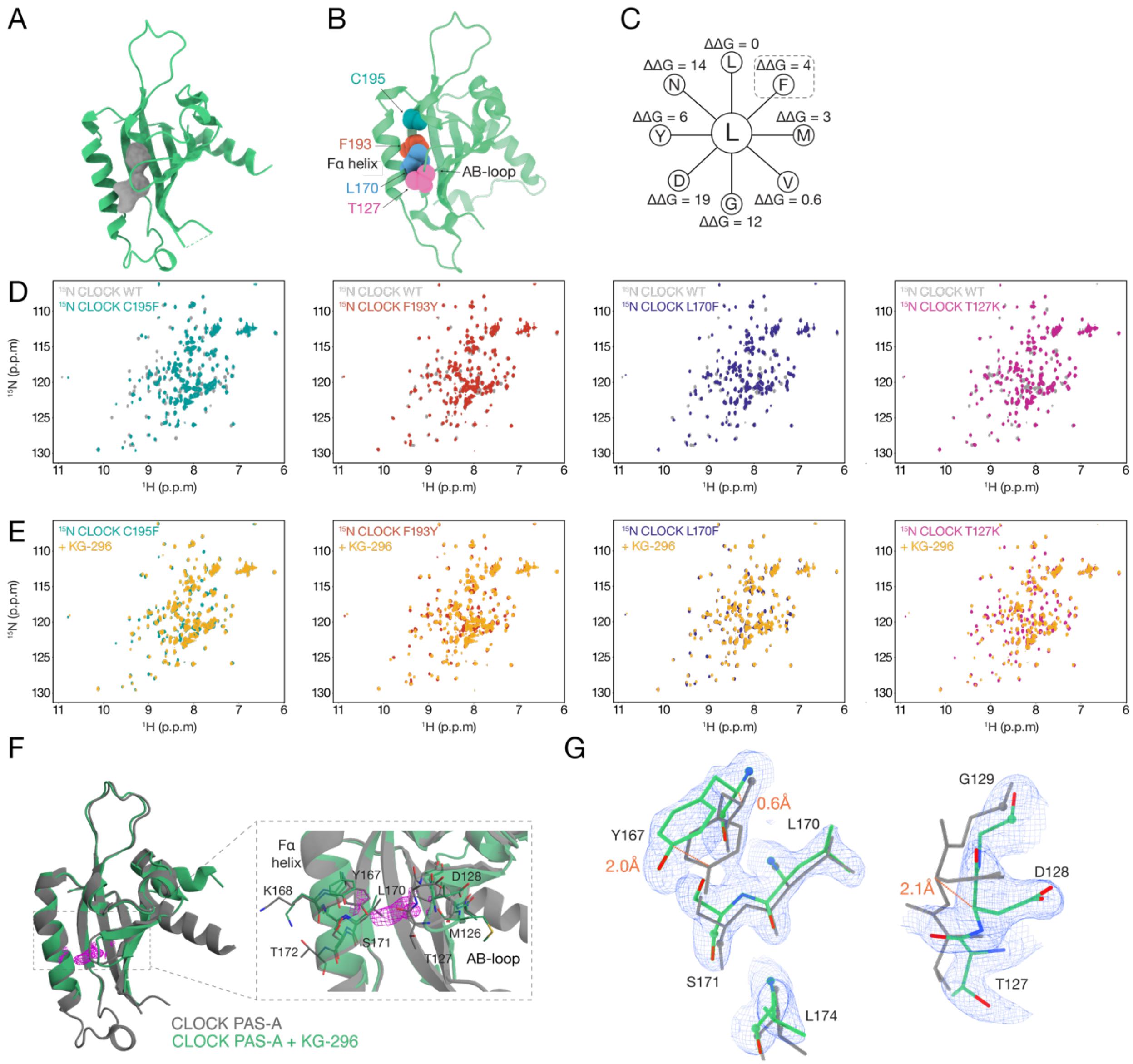

Figure S3: Screening mutants to disrupt KG-296 binding to CLOCK PAS-A. (A) CLOCK PAS-A (PDB 6QPJ, green) illustrating computationally mapped buried cavity (gray). **(B)** CLOCK PAS-A structure (PDB 6QPJ, green) highlighting location of mutants screened for KG-296 binding. **(C)** Pythia schematic showing some examples of L170 mutants and their ΔΔG scores in kcal/mol. **(D)** Overlay of ^{15}N-^{1}H HSQC of ^{15}N CLOCK PAS-A WT (gray) with ^{15}N labeled CLOCK PAS-A mutants, C195F, teal; F193Y, brown; L170F, dark blue; T127K, pink. **(E)** ^{15}N-^{1}H HSQC spectra of ^{15}N CLOCK PAS-A mutants with 500 µM KG-296, as colored in (D) for apo, yellow with ligand. **(F)** Structure overlay of apo CLOCK PAS-A (gray, PDB:6QPJ) with CLOCK PAS-A + KG296 (green) showing experimental density near KG-296 from a $2mF_o$-DF_c simulated-annealing composite omit map contoured at 1σ. **(G)** Zoom view of residues with change in backbone conformation with KG-296 incubation (green) relative to apo (gray).

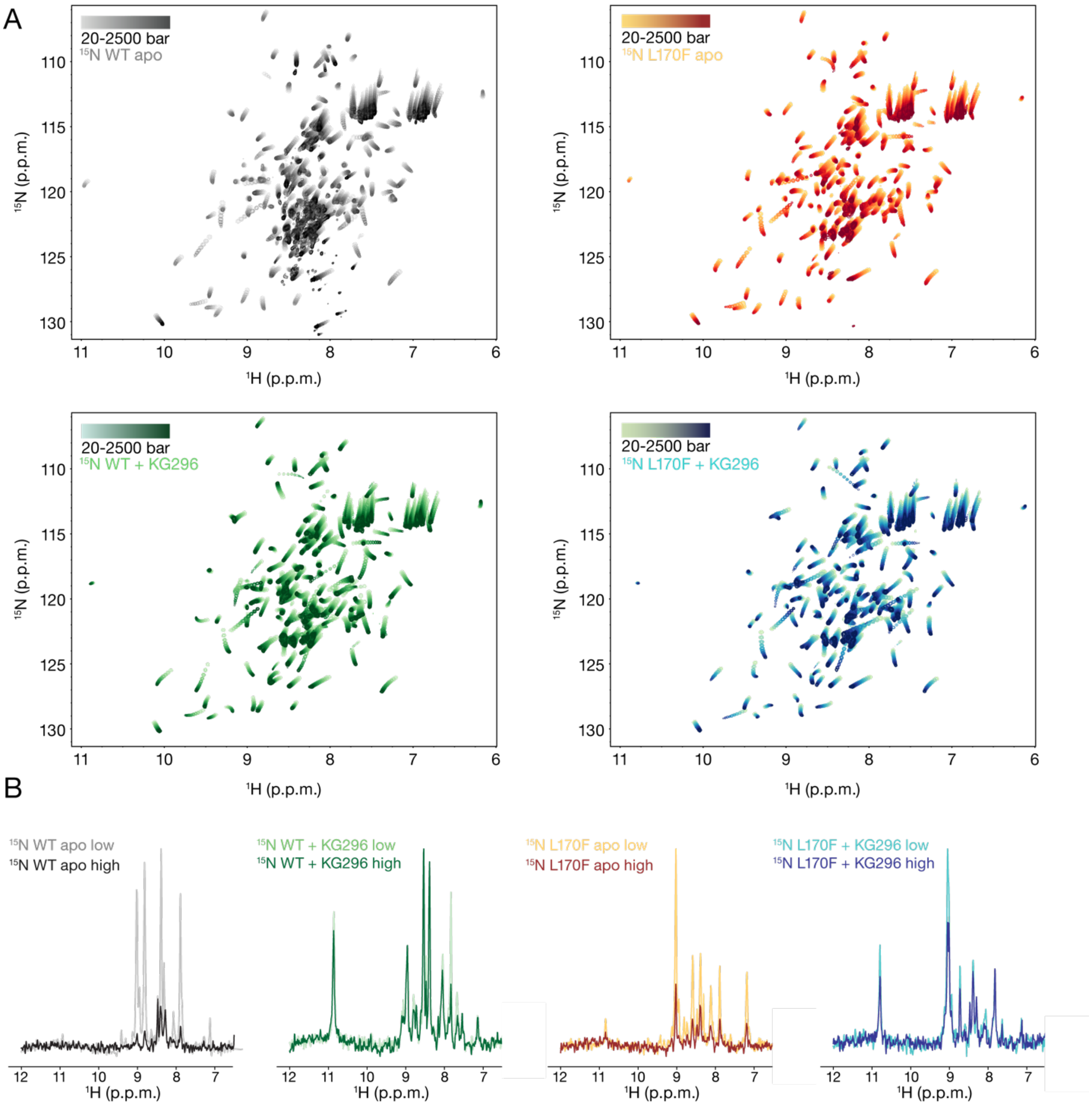

Figure S4: High-pressure NMR titration reveals changes in CLOCK PAS-A due to KG-296 and/or L170F mutation. (A) Overlays of ^{15}N-^{1}H HSQC spectra acquired under increasing pressure from 20-2500 bar of CLOCK PAS-A apo (gray to black, top left), CLOCK PAS-A + KG296 (light green to dark green, bottom left), CLOCK PAS-A L170F apo (yellow to maroon, right left), CLOCK PAS-A L170 + KG-296 (light turquoise to dark blue, bottom right). (B). Overlay of ^{1}H 1D traces extracted from ^{15}N-^{1}H HSQC spectra at ^{15}N = 118 ppm at 20 bar beginning of pressure titration point 250 bar (low) and at 20 bar after end of pressure titration point 2250 bar (high). WT apo (low, gray; high, black), CLOCK PAS-A + 1200 μM KG-296 (low, light green; high, dark green), L170F apo (low, yellow; high, maroon), L170F + 1200 μM KG-296 (low, turquoise; high, dark blue).

86 **Table S1.** X-ray crystallography data collection and refinement statistics

Data collection	hCLOCK PAS-A L170F
PDB ID	9PTN
Resolution range	38.92 - 1.75 (1.813 - 1.75)
Space group	P 1 21 1
Unit cell	45.85 45.2101 76.63 90 92.97 90
Total reflections	307651 (30689)
Unique reflections	31391 (3077)
Multiplicity	9.8 (9.9)
Completeness (%)	97.11 (97.31)
Mean I/sigma(I)	40.73 (5.08)
R-merge	0.5484 (0.7164)
CC1/2	0.792 (0.826)
Refinement statistics	
R-work	0.1987
R-free	0.2179
Number of non-hydrogen atoms	2335
macromolecules	2128
solvent	207
RMS(bonds)	0.007
RMS(angles)	1.03
Ramachandran favored/allowed (%)	100
Ramachandran outliers (%)	0.00
Average B-factor	23.59
macromolecules	22.89
solvent	30.71

87 Statistics for the highest-resolution shell are shown in parentheses.
